# Provision of Digital Primary Health Care Services: Overview of Reviews

**DOI:** 10.2196/53594

**Published:** 2024-10-29

**Authors:** Virgínia Maria Dalfior Fava, Luís Velez Lapão

**Affiliations:** 1 Centro de Estudos Estratégicos Antonio Ivo de Carvalho Fundação Oswaldo Cruz (Fiocruz) Ministério da Saúde Rio de Janeiro Brazil; 2 Intelligent Decision Support Systems Laboratory Research & Development Unit for Mechanical and Industrial Engineering (UNIDEMI) NOVA School of Science and Technology, Universidade Nova de Lisboa Caparica Portugal; 3 Laboratório Associado de Sistemas Inteligentes (LASI) Escola de Engenharia Universidade do Minho Guimarães Portugal; 4 WHO Collaborating Center on Health Workforce Policy and Planning Instituto de Higiene e Medicina Tropical Universidade NOVA de Lisboa Lisboa Portugal

**Keywords:** primary health care, digital health, implementation, health service quality, patients’ clinical conditions, digital skills, mobile phone

## Abstract

**Background:**

Digital health is a growing field, and many digital interventions have been implemented on a large scale since the COVID-19 pandemic, mainly in primary health care (PHC). The development of digital health interventions and their application in PHC are encouraged by the World Health Organization. The increased number of published scientific papers on this topic has resulted in an overwhelming amount of information, but there is no overview of reviews to summarize this evidence.

**Objective:**

This study aims to provide policy makers, health managers, and researchers with a summary of evidence on digital interventions used in PHC.

**Methods:**

This overview of reviews searched the Web of Science and MEDLINE databases for systematic and scoping reviews on assessments of digital technologies implemented in PHC published from January 2007 to March 2023. Only reviews that addressed digital interventions whose targets were real patients or health care providers (HCPs) were included.

**Results:**

A total of 236 records were identified from the search strategy, of which 42 (17.8%) full-text papers were selected for analysis, and 18 (7.6%) reviews met the eligibility criteria. In total, 61% (11/18) of the reviews focused their analysis on specific digital health interventions (client-to-provider telemedicine, provider-to-provider telemedicine, health worker decision support systems, systems for tracking patients’ health status, client participation and self-care platforms, and provision of education and training to health workers), and 39% (7/18) of the reviews focused on specific topics related to PHC (preventive care, chronic disease management, behavioral health disorders, the COVID-19 pandemic, multicomponent PHC interventions, and care coordination). Most studies in the included reviews agreed on barriers to implementation, such as software and apps developed without involving end users, the lack of training of HCPs and patients in digital technology use, and the lack of reimbursement and billing strategies for remote consultations. However, they showed several mixed results related to health service quality and patients’ clinical conditions and behavior changes.

**Conclusions:**

Research in digital health applied to PHC is still concentrated in high-income countries, mainly in North America and Europe. The mixed results related to health service quality and patients’ clinical conditions or behavior changes may have been caused by deficiencies in the process of implementing digital interventions. It is necessary to examine the entire impact pathway and the causal relationship among implementation, health service quality, and clinical condition outcomes to support the spread of digital health in PHC settings.

## Introduction

### Background

Digital health is a growing field combining medical informatics, health care monitoring, and new business models that involves delivering health services and information through the internet and related technologies. The goal is to use information and communications technology to improve health care on a local, regional, and global scale [1]. Although many digital health software and apps have been developed and made available since the 2000s, only since the COVID-19 pandemic did these digital interventions begin to be implemented on a large scale, mainly in primary health care (PHC), highlighting their importance for health care [2,3].

Many studies have demonstrated the contributions of information and communications technologies to enhance health care. Digital health interventions have been effective in improving health behavior, clinical assessment, treatment compliance, and coordination of care [4]. Positive impacts of digital health interventions have been observed regarding improvements in glycated hemoglobin levels in patients diagnosed with type 1 and type 2 diabetes mellitus and prediabetes [5]; greater medication adherence and physical activity, reduced cardiovascular risks, and better detection of arrhythmias in cardiovascular disease care [6]; general health behavior engagement, such as physical activity, dietary behaviors, medication adherence, and sun protection practices [7]; quality of life, psychological outcomes, and screening behaviors in patients with cancer [8]; and mental health [9].

The current relevance of this topic at a global level is demonstrated by the launch of the new Global Initiative on Digital Health by the World Health Organization (WHO) at the Health Ministers’ Meeting of the G20 Summit. The Global Initiative on Digital Health is a network of stakeholders to spread digital health, worldwide and within countries, through a comprehensive global ecosystem to promote country capacity and strengthen international cooperation in digital health [10]. The WHO has especially promoted the use of digital health in PHC settings. The Global Conference on Primary Health Care in Astana, Kazakhstan, in October 2018 encouraged the adoption of digital health in PHC settings to improve health care access and quality, patient safety, and care coordination in addition to empowering individuals and communities to take an active role in maintaining their overall health and well-being [11].

Digital technologies can improve PHC in many ways: integrating clinical support tools and referral systems into PHC to help coordinate care and ensure its continuity; supporting and building capacity among the health workforce; supporting patients in home settings through access to personalized information, appointment booking, and tools to manage their chronic conditions; making point-of-care diagnostic testing available for rapid analysis; and identifying risks and reducing harm to guarantee patient safety [12]. In addition, PHC has been critical for three main reasons: (1) it presents features that allow the health system to adapt and respond to a complex and rapidly changing world, (2) it emphasizes promotion and prevention with a people-centered approach that addresses the causes and risk factors of poor health, and (3) it has contributed to universal health coverage and the health-related Sustainable Development Goals. This is possible because PHC has an approach focused on comprehensive care, which includes promotive, protective, preventive, curative, rehabilitative, and palliative care throughout the life course, which addresses the determinants of health in a broad way (social, economic, and environmental factors, as well as individual characteristics and behavior) and seeks to empower individuals, families, and communities to optimize their health [13].

Recent bibliometric studies of scientific production on eHealth or digital health have confirmed the increase in publications on this topic in the last few years, mainly in North America and Europe but also in Australia, China, and India [14-17]. The increased number of published scientific papers on digital health has resulted in an overwhelming amount of information; however, most studies are not actual implementations in the real patient environment [18]. To make this information more accessible and usable, researchers have been summarizing it into review articles. They have also conducted overviews of reviews to address broader questions that go beyond the scope of individual reviews [19]. Policy makers play a crucial role in promoting the implementation of digital technologies in PHC by prioritizing public investment in building the physical infrastructure; deploying information systems and apps; developing a capable health workforce; ensuring a sound legal and regulatory environment; and improving governance, policy, standardization, and interoperability [12].

### Objectives

Studies that have conducted overviews of reviews on digital health have addressed many questions focused on technology implementation [20], impacts on health care services [21,22], specific clinical conditions such as cancer [8] and mental health [23], or specific types of digital interventions such as mobile apps [24]. However, there is no overview of reviews to inform policy makers, health managers, and researchers on digital intervention outcomes on PHC. Considering the importance of PHC for global health and the advances in research on the use of digital technologies in PHC settings, this overview of reviews aimed to summarize the evidence on digital interventions applied to PHC to inform policy makers, health managers, and researchers.

## Methods

### Search Strategy

An overview of reviews was conducted through an electronic literature search of review articles focused on digital technologies applied to PHC, following the PRISMA (Preferred Reporting Items for Systematic Reviews and Meta-Analyses) checklist [25] (Multimedia Appendix 1). We searched the Web of Science Core Collection and PubMed/MEDLINE databases from the National Library of Medicine. Unpublished and gray literature were not pursued.

The search was conducted in March 2023 and was based on a query with 2 parts (Multimedia Appendix 2): the first one covered topics referring to digital technologies applied to health (ie, *digital health*, *e-health*, *medical informatics*, *health informatics*, and *health IT*), and the second one included topics associated with PHC (ie, *primary care*, *primary healthcare*, and *primary health care*). The review articles had to be published since 2007 and written in English or Portuguese. Duplicated records were excluded.

### Eligibility Criteria

The included reviews were required to meet the eligibility criteria established by the population, intervention, comparator, outcome, and study design (PICOS) framework formulated for this study. Textbox 1 outlines the eligibility criteria according to the 5 PICOS components [26].

The digital interventions analyzed by the included reviews were classified according to WHO recommendations [27] into 8 types of interventions, described in Textbox 2.

In total, 2 authors screened the titles and abstracts and excluded studies that did not meet the eligibility criteria. Disagreements were resolved through discussion and consensus between the authors. Full-text versions of the remaining articles were obtained and screened, and papers were also excluded if digital health was not integrated into PHC services or if other settings were included without results presented by level of care. The reasons for exclusion were recorded during the 2 steps (title or abstract and full-text screening).

**Population, intervention, comparator, outcome, and study design (PICOS) framework formulated for this study.**

**PICOS component and eligibility criteria**

- Participants: any patient or health care provider in primary health care
- Intervention: digital interventions implemented in primary health care settings whose targets were real patients or health workers
- Comparisons: no treatment, treatment as usual, or different types of digital technologies
- Outcomes: implementation, health service quality, or patients’ clinical conditions or behavior change outcomes
- Study design: systematic or scoping reviews

**Types of digital health interventions selected for this study.**

**Digital health interventions**

- Client-to-provider telemedicine: delivery of health care services in which clients or patients and health workers are separated by distance
- Provider-to-provider telemedicine: delivery of health care services in which ≥2 health workers are separated by distance
- Targeted client communication: transmission of customized health information for different audience segments based on health status or demographic categories
- Health worker decision support: digitized job aids that combine an individual’s health information with the health worker’s knowledge and clinical protocols to assist health workers in making diagnosis and treatment decisions
- Tracking of patients’ health status: digitized records used by health workers to capture and store health information on clients or patients to follow up on their health status and services received
- Provision of education and training to health workers: the management and provision of education and training content in digital form for health professionals, which does not need to be used at the point of care
- Client participation and self-care: digitized records used by patients to capture and monitor their health information and other digital tools for patient communication (client-to-client communication, access by the client to their own medical records, self-monitoring of health or diagnostic data by the client, and active data capture or documentation by the client)
- Laboratory and diagnostics imaging management: digital approaches to manage and exchange laboratory and diagnostic orders and results (transmit diagnostic results to HCPs, transmit and track diagnostic orders, and capture diagnostic results from digital devices)

### Data Extraction and Synthesis

The data were managed and stored using Zotero (Corporation for Digital Scholarship) and Excel (Microsoft Corp). Extracted data used to categorize review papers included publication year, authors, title, journal information (title and ISSN), abstract, and language. Additional information was extracted independently by 2 authors from each review paper, including study design, number of studies included, number of databases searched, classification of digital health interventions according to the WHO recommendations [27], intervention target, outcome category according to the PICOS framework, and a summary of the results.

### Risk-of-Bias Assessment

The quality of the evidence in responding to the research questions was assessed independently by 2 authors using the Joanna Briggs Institute Critical Appraisal Checklist for Systematic Reviews tool [28]. The 2 authors discussed the results of the quality appraisal, reaching a consensus in case of any divergence. Question 9 was not assessed because all the reviews summarized qualitative evidence.

Some criteria in the risk-of-bias assessment of scoping reviews were adapted considering the guidelines for preparing this type of study [29]. Question 1 was considered met if the review included clear and explicit statements about the objectives even if it did not explicitly present a research question. Questions 5 and 6 were not evaluated as conducting a critical appraisal of the included sources of evidence is not mandatory.

### Data Analysis

A narrative synthesis of the main results from the included reviews was conducted to identify the main types of digital technologies applied to PHC based on the classification of digital health interventions proposed by the WHO [27], which health problems and conditions were addressed, and the results of their implementation from the outcome categories in Textbox 1 (implementation outcomes, health service outcomes, and patients’ clinical conditions and behavior change outcomes).

## Results

### Search Results

**Figure 1:**
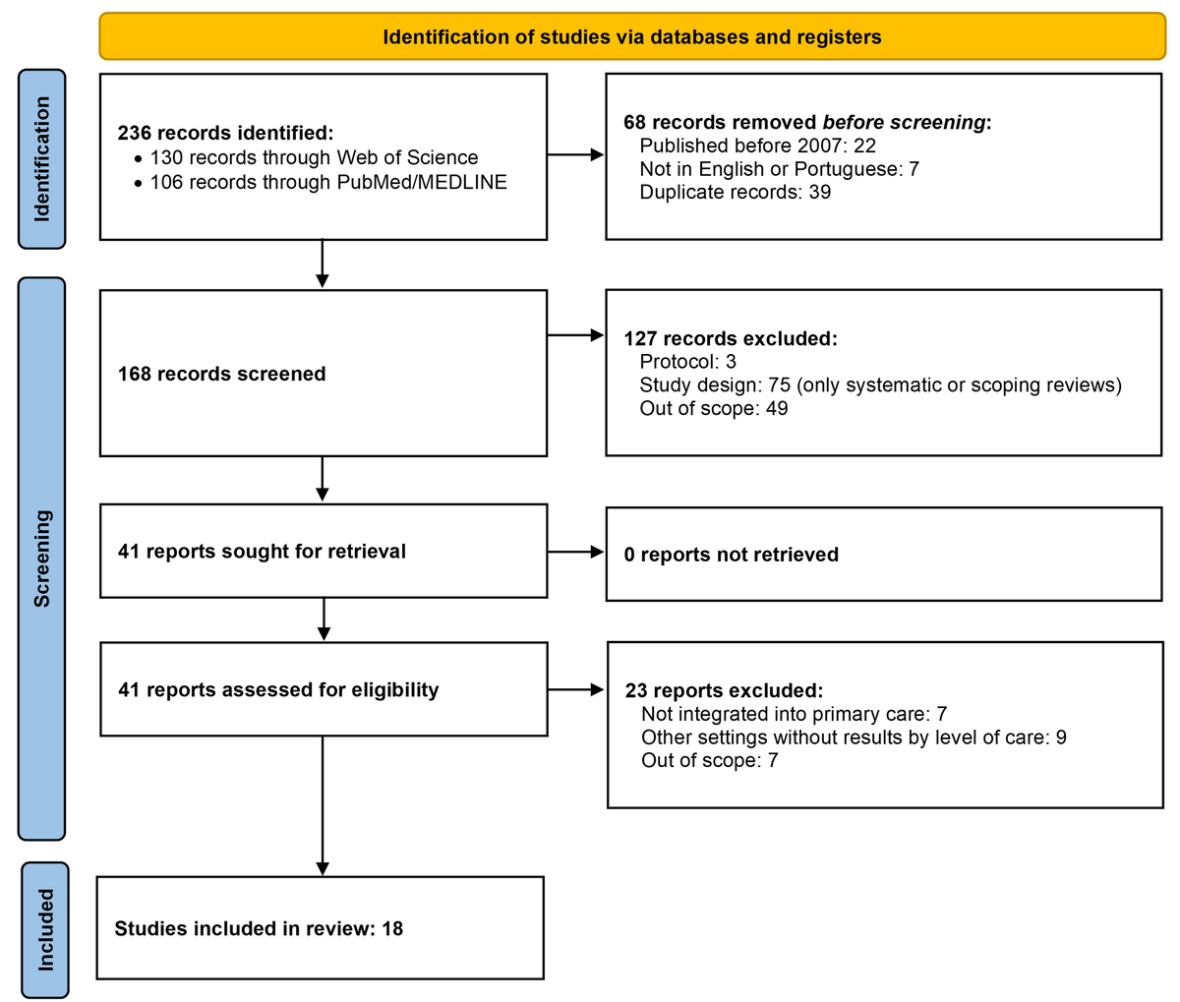
PRISMA (Preferred Reporting Items for Systematic Reviews and Meta-Analyses) flow diagram.

A total of 236 records were identified from the search strategy, of which 68 (28.8%) were excluded before analysis because of the publication date or language or for being duplicates. In the title and abstract screening, 75% (126/168) of the records were excluded because of the eligibility criteria. All 42 remaining integral text records were retrieved, and 24 (57%) were excluded after screening. The remaining 18 reviews met the eligibility criteria and were included in the synthesis. Figure 1 shows the PRISMA flow diagram for the total articles assessed.

### Review Characteristics

Table 1 presents the general characteristics of the reviews, including publication year, study design, number of studies included, number of databases searched, classification of digital health interventions, intervention target, and outcome category. All studies were published in English.

All reviews were published from 2015 onward, with 67% (12/18) published between 2021 and 2023. Of the 18 reviews, 11 (61%) were systematic reviews, and 7 (39%) were scoping reviews. A total of 72% (13/18) of the studies searched for articles in up to 5 databases. The scoping reviews included an average of 35.3 (SD 11.7) reports (outliers excluded: 241), and the systematic reviews included an average of 13.0 reports (outliers excluded: 43 and 48).

Table 2 provides the risk-of-bias assessment details. All systematic reviews except for one (10/11, 91%) presented good methodological quality, meeting at least 8 of the 10 criteria analyzed. Although all systematic reviews conducted critical appraisal to assess the included studies, that analysis was carried out by ≥2 independent reviewers only in 55% (6/11) of them. Similarly, most scoping reviews (5/7, 71%) presented a good methodological approach. The least attended item refers to the appropriate extraction of data, which was not always carried out by 2 independent researchers.

**Table 1:** General characteristics of the included reviews.

Review	Year	Study design	Databases, n	Included studies, n	Digital health intervention	Outcome categories
Hickson et al [30]	2015	Scoping review	2	24	Client-to-provider telemedicine	Implementation; health services
Beheshti et al [31]	2022	Scoping review	4	43	Client-to-provider telemedicine	Implementation; clinical conditions or behavior changes
Kuo and Dang [32]	2016	Systematic review	1	11	Client-to-provider telemedicine; client participation and self-care	Clinical conditions or behavior changes
Liddy et al [33]	2019	Systematic review	4	43	Provider-to-provider telemedicine	Implementation; health services; clinical conditions or behavior changes
Meunier et al [34]	2023	Systematic review	5	48	Health worker decision support	Implementation
El Asmar et al [35]	2021	Systematic review	6	8	Health worker decision support	Clinical conditions or behavior changes
Agarwal et al [36]	2021	Systematic review	7	8	Health worker decision support	Implementation; health services; clinical conditions or behavior changes
Peyroteo et al [18]	2021	Systematic review	2	18	Tracking of patients’ health status	Implementation
Tuan Soh et al [37]	2022	Systematic review	5	13	Client participation and self-care	Implementation
Andrikopoulou et al [38]	2019	Systematic review	7	15	Client participation and self-care	Clinical conditions or behavior changes
Kyaw et al [39]	2019	Systematic review	9	8	Provision of education and training to health workers	Health services; clinical conditions or behavior changes
Willis et al [40]	2022	Scoping review	3	241	Client-to-provider telemedicine; targeted client communication; health worker decision support; tracking of patients’ health status; client participation and self-care	Implementation; health services
Xiong et al [41]	2023	Scoping review	3	52	Client-to-provider telemedicine; targeted client communication; health worker decision support; client participation and self-care; provision of education and training to health workers; laboratory and diagnostics imaging management	Health services; clinical conditions or behavior changes
Moon et al [42]	2022	Scoping review	4	20	Client-to-provider telemedicine; targeted client communication; health worker decision support; tracking of patients’ health status; client participation and self-care; provision of education and training to health workers	Implementation; clinical conditions or behavior changes
Jonnagaddala et al [43]	2021	Scoping review	4	29	Client-to-provider telemedicine; client participation and self-care; provision of education and training to health workers	Implementation
Silva et al [44]	2022	Scoping review	17	44	Client-to-provider telemedicine; targeted client communication; client participation and self-care; provision of education and training to health workers	Implementation; health services
Jimenez et al [45]	2021	Systematic review	2	14	Client-to-provider telemedicine; provider-to-provider telemedicine; targeted client communication; health worker decision support; tracking of patients’ health status; client participation and self-care	Implementation; clinical conditions or behavior changes
Maillet et al [46]	2018	Systematic review	5	22	Health worker decision support; tracking of patients’ health status; client participation and self-care; laboratory and diagnostics imaging management	Implementation; health services

**Table 2:** The methodological quality of the reviews based on the Joanna Briggs Institute Critical Appraisal Checklist.

			Q1^a^		Q2^b^		Q3^c^		Q4^d^		Q5^e^		Q6^f^		Q7^g^		Q8^h^		Q9^i^		Q10^j^		Q11^k^
**Systematic reviews**																							
	Kuo and Dang [32]	Yes		No		Yes		No		Yes		No		No		Yes		—^l^		Yes		Yes	
	Liddy et al [33]	Yes		Yes		Yes		Yes		Yes		No		Yes		Yes		—		Yes		No	
	Meunier et al [34]	Yes		Yes		Yes		Yes		Yes		Yes		Yes		No		—		Yes		Yes	
	El Asmar et al [35]	Yes		Yes		Yes		Yes		Yes		Yes		Yes		Yes		—		Yes		Yes	
	Agarwal et al [36]	Yes		Yes		Yes		Yes		Yes		Yes		Yes		Yes		—		Yes		Yes	
	Peyroteo et al [18]	Yes		Yes		Yes		Yes		Yes		No		Yes		Yes		—		Yes		Yes	
	Tuan Soh et al [37]	Yes		Yes		Yes		Yes		Yes		Yes		No		Yes		—		Yes		Yes	
	Andrikopoulou et al [38]	Yes		Yes		Yes		Yes		Yes		Yes		Yes		Yes		—		Yes		Yes	
	Kyaw et al [39]	Yes		Yes		Yes		Yes		Yes		Yes		Yes		Yes		—		Yes		Yes	
	Jimenez et al [45]	Yes		Yes		Yes		No		Yes		No		Yes		Yes		—		Yes		Yes	
	Maillet et al [46]	Yes		Yes		Yes		Yes		Yes		No		No		Yes		—		Yes		Yes	
**Scoping reviews**																							
	Hickson et al [30]	Yes		Yes		Yes		No		—		—		No		Yes		—		Yes		Yes	
	Beheshti et al [31]	Yes		No		Yes		Yes		—		—		Yes		No		—		Yes		Yes	
	Willis et al [40]	Yes		Yes		Yes		Yes		—		—		Yes		Yes		—		Yes		Yes	
	Xiong et al [41]	Yes		Yes		Yes		Yes		—		—		Yes		Yes		—		Yes		Yes	
	Moon et al [42]	Yes		Yes		Yes		Yes		—		—		No		Yes		—		Yes		Yes	
	Jonnagaddala et al [43]	Yes		Yes		Yes		Yes		—		—		Yes		Yes		—		Yes		Yes	
	Silva et al [44]	Yes		Yes		Yes		Yes		—		—		Yes		Yes		—		Yes		Yes	

^a^Question 1: Is the review question clearly and explicitly stated?

^b^Question 2: Were the inclusion criteria appropriate for the review question?

^c^Question 3: Was the search strategy appropriate?

^d^Question 4: Were the sources and resources used to search for studies adequate?

^e^Question 5: Were the criteria for appraising studies appropriate?

^f^Question 6: Was critical appraisal conducted by ≥2 reviewers independently?

^g^Question 7: Were there methods to minimize errors in data extraction?

^h^Question 8: Were the methods used to combine studies appropriate?

^i^Question 9: Was the likelihood of publication bias assessed?

^j^Question 10: Were recommendations for policy or practice supported by the reported data?

^k^Question 11: Were the specific directives for new research appropriate?

^l^Not applicable.

### Types of Digital Health Interventions Applied to PHC

#### Overview

A total of 11 review papers analyzed 1 specific type of digital health intervention applied to PHC—(1) 3 (27%) reviewed client-to-provider telemedicine interventions, (2) 1 (9%) reviewed provider-to-provider telemedicine interventions, (3) 3 (27%) reviewed health worker decision support interventions, (4) 1 (9%) reviewed interventions tracking patients’ health status, (5) 2 (18%) reviewed client participation and self-care interventions, and (6) 1 (9%) reviewed interventions providing education and training to health workers (Textbox 3).

**Review papers according to the type of digital health intervention analyzed.**

**Client-to-provider telemedicine**

- Hickson et al [30]
- Beheshti et al [31]
- Kuo and Dang [32]

**Provider-to-provider telemedicine**

- Liddy et al [33]

**Health worker decision support**

- Meunier et al [34]
- El Asmar et al [35]
- Agarwal et al [36]

**Tracking patients’ health status**

- Peyroteo et al [18]

**Client participation and self-care interventions**

- Tuan Soh et al [37]
- Andrikopoulou et al [38]

**Provision of education and training to health workers**

- Kyaw et al [39]

#### Client-to-Provider Telemedicine

A total of 29% (2/7) of the scoping reviews analyzed the literature on the state of client-to-provider telemedicine with a focus on nonurgent PHC practice. One review focused on web-based consultations using mobile devices [30], and the other included all remote health services delivered via real-time communication between the patient and the HCP [31]. Both reviews agreed that this kind of intervention allows for patient follow-up and clinical condition monitoring and aims to provide quick and easy access to meet medical needs.

In addition, a systematic review examined the evidence supporting the use of secure messages associated with other electronic health record (EHR) resources, such as patient portals and personal health records (PHRs; client participation and self-care interventions), focused on diabetes management. Secure messaging was defined as “any electronic communication between a HCP and patient that ensures only those parties can access the communication” [32].

Beheshti et al [31] showed that telehealth has spread in many countries, including papers that analyzed interventions in Europe (half of them, ie, 55.8% from the United Kingdom), United States (34.9%), Asia (2 papers), and Brazil and Zambia (1 paper each). Most studies analyzed in this review focused on investigating the effects of these services on rural and underserved areas.

However, the concentration of studies in Europe and the United States may be associated with the various implementation barriers cited by Hickson et al [30] and Beheshti et al [31]. As obstacles to the implementation of client-to-provider interventions, they cited infrastructure barriers such as lack of security to transmit confidential information, low bandwidth, unsuitable networks, low signal quality, and bad image quality; system barriers such as the lack of integration with electronic medical records, workflow, and other existing systems; legal barriers such as professional regulation when patients and HCPs are in different states or countries; and billing and reimbursement barriers to guarantee the monetary incentives for HCPs to deliver remote health services. These studies also presented patient barriers, such as confidentiality and privacy concerns and the demand for supervisor support, and HCP barriers, such as reluctance toward new approaches to health care delivery, concerns about the absence of patient health literacy, the perception that telehealth technology is unintuitive and inflexible, and the fear of increased workload.

Despite the implementation difficulties, Hickson et al [30] presented studies that showed improvements in health service outcomes. Using remote health services through mobile devices resulted in cost savings for acute care delivery by decreasing in-office spending per member per month and by preventing face-to-face visits in settings that applied web-based consultation reimbursements to a fee-for-service model of patient payment. Beheshti et al [31] presented only 1 study that indicated clinical condition improvements in patients with diabetes with lower blood sugar levels using telemedicine, in addition to other benefits such as better access to health care, less waste of time, and a high degree of satisfaction with health care. In addition, Kuo and Dang [32] reviewed 11 studies, of which 7 suggested a significant improvement in the glycemic control of patients with diabetes (primary outcome) resulting from the use of secure messaging, but the impact on other outcomes such as cholesterol and blood pressure was inconsistent. A total of 10 of the studies included in this review were conducted in North America.

The 2 scoping reviews also specified that there are several communication tools for delivering remote health care but asynchronous technologies (eg, emails and SMS text messages) seemed to be supplementary and did not reduce the frequency of face-to-face visits. The synchronous form is the most dominant approach for delivering telehealth services (eg, telephone calls, videoconferences, and teleconferences). Finally, despite the identified benefits of client-to-provider telemedicine interventions cited by these 2 reviews, such as cost reduction, decreased face-to-face visits, patients’ time savings, and health care access and clinical condition improvements, the 2 scoping reviews pointed out that more research is necessary to replicate and expand findings on access to health care and patients’ clinical conditions to identify impacts on workflow efficiency and cost-effectiveness depending on billing and reimbursement strategies [30,31].

#### Provider-to-Provider Telemedicine

A systematic review examined the impact of electronic consultations on the delivery of care, applying the quadruple aim framework that analyzes the effects on population health, the experience of receiving care (patients), the experience of providing care (HCPs), and per-capita costs [33]. This review defined an electronic consultation (e-consult) as an electronic communication tool that allows PHC HCPs to obtain a specialist consultant’s expert opinion promptly.

Most studies included in the systematic review were from the United States (19/43, 44%) and Canada (12/43, 28%). In terms of implementation outcomes, e-consults had sustained use and spread, high adoption, and little pushback from HCPs. The range of specialties accessed by PHC HCPs through e-consults expanded beyond dermatology, including multispecialty services that comprise endocrinology, hematology, cardiology, gastroenterology, and neurology.

The results presented by this systematic review showed that most PHC HCPs reported being satisfied with e-consults, they intended to use this technology in the future because their questions were answered, and the specialty support was conclusive without impact on the workload. However, there were still some challenges, such as unclear directions from specialists to PHC HCPs, lack of information or pertinent questions delivered to specialists by PHC HCPs, lack of patient follow-up by the specialists, and a potential increase in workload for specialists.

Regarding health service outcomes, Liddy et al [33] pointed out that e-consults are effective in faster access to specialist advice and substantial avoidance of face-to-face visits and showed that the waiting time for a specialist response was less than that for traditional referrals. Related to patients’ clinical condition effects, they indicated a potential for reducing adverse cardiovascular outcomes, but there was a potential for harm due to the lack of patient follow-up.

The authors concluded that there are gaps in the evidence on the implementation of provider-to-provider digital interventions relative to specialists’ workload and patients’ perceptions. Furthermore, the divergent findings related to impacts on cost-effectiveness and clinical conditions indicate the need for more research in this area.

#### Health Worker Decision Support

In total, 27% (3/11) of the systematic reviews analyzed health worker decision support interventions [34-36]. All reviews examined aspects related to computerized clinical decision support systems (CDSSs), any software designed to offer patient-specific assessments and recommendations to the HCP based on patient data from EHRs and evidence-based clinical practice guidelines. One review sought to identify and quantify the barriers to and facilitators of the use of CDSSs by PHC HCPs [34]. The second one provided a summary of the effects of CDSS use on the clinical outcomes of adult patients with chronic diseases managed in PHC [35]. The third one focused their analysis on CDSSs accessible via mobile devices by PHC HCPs [36]. Most studies included in these 3 reviews were from North America (United States and Canada), Europe, and Australia, with a few studies from Asia, Africa, and South and Central America.

Meunier et al [34] assessed the implementation of CDSSs using the human, organization, technology, net benefits framework, which comprises the interdependent human, organizational, and technological factors related to health information system adoption in PHC. In the human category, they found that the HCPs’ satisfaction and their perceived usefulness of the CDSS recommendations could facilitate the use and adoption of this digital technology. However, when the CDSS offers information overload and recommendations conflicting with HCPs’ expertise, it might result in the HCPs’ resistance, reluctance, and negative attitude toward CDSS use. Furthermore, providing training in computer skills and software use could be a human facilitator. The main factors related to the organizational category are workflow and teamwork. When the CDSS is integrated into the EHR and the clinical workflow, producing quality reports and reducing workload through the expansion of the skill sets and roles of other health professionals in assisting physicians, it could facilitate the use of this digital technology. Conversely, the workflow disruption caused by CDSS implementation, demanding more teamwork and increasing the workload, might become a barrier. Finally, in the technological category, the ease of use, visual layout, usefulness of system features and functions, full integration with EHR databases (including data on specialty care), and technical support were pointed out as factors that could influence the use of the CDSS. However, the lack of computers and tablets and the slowness and poor performance of the software were identified as barriers to CDSS use. Agarwal et al [36] sought to verify the HCPs’ acceptability of and satisfaction with mobile CDSSs, but they did not find direct evidence of this implementation outcome.

Related to health service outcomes, Agarwal et al [36] reviewed papers that analyzed the HCPs’ adherence to recommended practices, guidelines, or protocols and the time between clinical recommendation presentation and appropriate management only for mobile CDSSs. The results were inconclusive for the first outcome because the certainty of evidence was very low, and no evidence was identified for the second outcome.

El Asmar et al [35] and Agarwal et al [36] searched for CDSS use impact on patients’ clinical conditions or behavior changes. The quality of evidence of the studies included in both reviews was rated as moderate, low, or very low. El Asmar et al [35] focused on clinical outcomes of the management of chronic diseases and presented data on diabetes mellitus, asthma, angina, hyperlipidemia, and hypertension. Most results indicated no statistically significant differences between CDSS intervention and control participants (usual care or baseline), and it is important to highlight that the study that showed a statistically significant impact on blood pressure between the intervention and control groups revealed extensive training for participants and widespread use by HCPs. Agarwal et al [36] reported similar results for clinical outcomes of chronic disease management—mobile CDSS use made little or no difference in clinical conditions for people with high cardiovascular disease risk, poorly controlled diabetes, or hyperlipidemia. The authors still presented inconclusive results for the impact on maternal and neonatal health outcomes because of the uncertainty of the evidence. However, they pointed out that the use of mobile CDSSs by community workers may increase medication adherence in people with high cardiovascular disease risk. However, the use of this technology by physicians made little or no difference in people with poorly controlled diabetes.

#### Tracking Patients’ Health Status

A systematic review analyzed the literature on remote health monitoring systems for chronic disease management in PHC settings [18]. This type of technology is classified as an intervention tracking patients’ health status. The included studies focused on interventions that automatically monitor patient indicators (sensors or wearables) continuously or those in which patients must collect and transmit personal electronic health information in real time or in an asynchronous way. Most studies included were from Europe and the Americas, with a few studies from Asia and Africa.

The studies included in this review indicated that there is a need for more research in this area. Most of the articles presented implementation processes as a proof of concept, pilot study, or clinical trial without evidence of the impact on actual health services or patients’ clinical conditions or behavior change outcomes. The authors showed some barriers to the intervention’s large-scale implementation, such as the low involvement of health workers, patients, and other stakeholders in the tool design and implementation. The low adherence by HCPs was explained by the lack of technology integration into the existing workflow, limited integration into the information systems already used, and the divergences between the data presented by the remote monitoring systems and the HCPs’ expertise in chronic disease management.

Peyroteo et al [18] concluded that it is necessary to fill in the implementation gaps mentioned to ensure the correct and large-scale use of the digital tool. This is the only way to assess the impacts of interventions tracking patients’ health status on health services and patients’ clinical conditions or behavior change outcomes.

#### Client Participation and Self-Care

In total, 18% (2/11) of the systematic reviews examined client participation and self-care interventions in PHC. One aimed to identify the benefits of using electronic PHRs based mainly on studies that assessed the patients’ and HCPs’ perceptions of the implementation of this technology [37]. The other review focused on the PHR features that improve medication adherence in the adult population with chronic diseases [38]. Both reviews defined PHRs as a tool that provides patients with their health care and personal medical information, and most included studies were conducted in North America or Europe.

Regarding implementation outcomes, Tuan Soh et al [37] found evidence indicating users’ favorable opinions on using PHR systems. The positive effects pointed out by these authors were on patient empowerment; improvement in self-care management, communication, and the relationship between patients and HCPs; and improvement in quality of care by supporting shared decision-making based on shared data, responsibility for health information that could be updated anytime by the patient, in addition to supporting interventions tracking patients’ health status and promoting continuity between the different levels of care. However, the authors indicated that more research is needed to understand structural, organizational, and policy barriers to the implementation of PHR systems and the challenges to ensure data accuracy and reliability.

Andrikopoulou et al [38] focused their review on the analysis of one specific patient behavior change outcome: medication adherence. In total, 13 of the 15 studies included reported positive impacts on medication adherence, and only 2 found no difference. This positive tendency was found despite the heterogeneity in participants’ demographics, study location, chronic disease, or medication adherence measurement method. The authors supposed that these results were due to specific PHR features such as reminders, feedback, and alerts on medication management as all studies included at least one of these features. Finally, they highlighted some gaps that demand more research, such as considering the particularities of patients with multimorbidity or polypharmacy when designing PHR resources to guarantee medication adherence.

#### Provision of Education and Training for Health Workers

A systematic review analyzed the provision of education and training to health workers on antibiotic management in PHC to summarize evidence on the effectiveness of digital education compared to traditional education for improving clinical practice [39]. For this reason, there were no reports of implementation outcomes, such as human, organizational, or technological barriers and facilitators. All included studies were from Europe and North America, except for one that was conducted in China.

Regarding health services, the authors presented results of specific knowledge outcomes, clinical practice improvements, and physicians’ attitudes toward the interventions. They found that digital education (mobile phone or PDA device with internet access) may improve knowledge scores measured using questionnaires compared to traditional education. Some forms of digital education (eg, email with feedback and suggestions and web-based learning resources) showed an impact on reducing antibiotic prescribing or dispensing rates in 4 studies included in the review, and another 2 studies indicated no difference between digital and traditional education when using SMS text messaging or web-based blended education. These results seemed to be more relevant when digital education was combined with web-based training on point-of-care diagnostic tests and enhanced communication skills. Finally, one study reported that almost all physicians were satisfied with the intervention and wanted to continue receiving clinical recommendations about antibiotic prescriptions.

In total, 4 studies included in the review by Kyaw et al [39] assessed patients’ clinical conditions measured using reconsultation rate, probability of consultation for a new respiratory tract infection, hospital referral, and postintervention hospital admission rate. In summary, these studies reported little or no difference between digital and traditional education. However, it can be assumed that the real impact of digital education on antibiotic prescription may be on antibiotic resistance.

### Health Issues Addressed by Digital Health Interventions in PHC Settings

#### Overview

A total of 28% (5/18) of the review papers searched for all types of digital interventions applied to six specific health issues in PHC settings: (1) preventive care, (2) chronic disease management, (3) behavioral health disorders, (4) the COVID-19 pandemic, (5) multicomponent PHC interventions, and (6) care coordination (Textbox 4).

**Review papers according to the type of digital health intervention and the topic related to primary health care analyzed.**

**Preventive care**

- Willis et al [40]

**Chronic disease management**

- Xiong et al [41]

**Behavioral health disorders**

- Moon et al [42]

**COVID-19 pandemic**

- Jonnagaddala et al [43]
- Silva et al [44]

**Multicomponent primary health care interventions**

- Jimenez et al [45]

**Care coordination**

- Maillet et al [46]

#### Preventive Care

A scoping review examined the digital interventions used in the US PHC services to enhance and support the delivery of primary, secondary, tertiary, and quaternary preventive care [40]. Most digital interventions identified in this review focused on electronic medical records, with alerts to help health professionals make appropriate clinical decisions (interventions for tracking patients’ health status and health worker decision support), or mobile interventions to improve patients’ self-care (client participation and self-care interventions). In addition to these types, the authors identified the use of client-to-provider telemedicine and targeted client communication interventions to promote preventive care.

Most studies included in this review showed statistically significant improvements in primary prevention health services and implementation outcomes (adolescent and adult vaccination); secondary prevention health service outcomes (screening rates for colorectal cancer, hepatitis C virus, and osteoporosis but not for colonoscopy); and tertiary prevention health service and clinical condition or behavior change outcomes for managing diabetes, hypertension, asthma, obesity, cardiovascular disease, hyperlipidemia, and mental conditions. There were no studies included on digital interventions in quaternary prevention. Finally, the authors concluded that digital interventions enhanced PHC by augmenting the comprehensiveness of the care provided but there are still gaps in the evaluations of mobile interventions [40].

#### Chronic Disease Management

A scoping review summarized the evidence on digital interventions for chronic disease management in PHC in low- and middle-income countries (10 studies from South America, 9 from Asia, and 2 from Africa) [41]. The review identified digital interventions of almost all types: client-to-provider telemedicine, targeted client communication, health worker decision support, client participation and self-care, provision of education and training to health workers, and laboratory and diagnostics imaging management. However, most digital health interventions still used SMS text messaging and smartphone apps for communication, making them easy to use and affordable as they only required cell phones and internet access.

This review indicated consistent positive results for implementation outcomes, such as improvements in accessibility, patient perception of the health service delivery, HCPs’ capabilities, and better cost-effectiveness. However, the clinical conditions and behavior change outcomes were highly mixed, with positive, negative, neutral, or nonsignificant results. In addition, the authors found that communication-related digital interventions (client-to-provider telemedicine and targeted client communication interventions) produced more consistent improvements in clinical conditions or behavioral and implementation outcomes but interventions focused on HCPs’ capacities (interventions for health worker decision support and provision of education and training to health workers) resulted in substantially mixed clinical condition or behavior change outcomes. These authors defend that research with a larger number of participants followed over a longer time may result in more consistent evidence of the impact of digital interventions [41].

#### Behavioral Health Disorders

A scoping review was conducted to gather and analyze research on how digital health services can enhance the collaborative care model, which addresses behavioral health disorders in PHC settings [42]. The studies included presented digital health interventions of different types: client-to-provider telemedicine, targeted client communication, health worker decision support, tracking of patients’ health status, client participation and self-care, and provision of education and training to health workers.

Regarding implementation outcomes, both patients and HCPs had positive perceptions of these digital interventions, which facilitated the communication between users and freed up HCP time for patients. Despite these positive outcomes, privacy and workload concerns arose when the technology was not fully integrated into the clinical workflow, resulting in low user engagement and high dropout rates. The improvements in clinical conditions or behavior change outcomes presented in the included studies were modest, partly due to methodological factors such as small sample sizes and different control groups [42].

#### COVID-19 Pandemic

A total of 29% (2/7) of the scoping reviews explored the use of digital interventions in PHC settings during the COVID-19 pandemic. One focused on the Australian PHC response to the pandemic and found interventions on client-to-provider telemedicine, client participation and self-care, and education and training for health workers [43]. The other was a more extensive review including 18 studies conducted in North America, 14 conducted in Europe, 4 conducted in South America, 4 conducted in Asia, and 4 conducted in Oceania; they identified targeted client communication interventions in addition to the digital technologies found in the other review [44]. Both reviews concluded that these digital technologies were implemented to manage COVID-19 as well as other common diseases in PHC settings.

The 2 reviews emphasized that the key facilitators in implementing digital technologies during the COVID-19 pandemic were providing technology training and support for HCPs; offering validated and reliable technologies with a user-centered design that were suitable for the local context; and ensuring user security, acceptability, and satisfaction. However, there were implementation barriers, such as lack of digital facilities in remote and rural areas, safety and privacy concerns, lack of planning for health services and clinical protocols when using digital tools, and the need for new funding arrangements that reimburse for remote services. Although digital technologies during the pandemic had particular importance for chronic condition management, reducing the number of referrals to specialties and hospitalizations and speeding up screening in cases of clinical changes through remote tracking, these tools were not always effective in identifying complex or urgent demands and assisting older adults and children [43,44].

According to Silva et al [44], digital health emerged as the sole substitute for PHC during the COVID-19 pandemic. However, the decrease in the quality of health care could be attributed to the hasty implementation of remote services in not properly equipped facilities and by health professionals who were under immense pressure.

#### Multicomponent PHC Interventions

A systematic review explored how digital health has enhanced multicomponent PHC interventions, defined as initiatives comprised of several features aimed at enhancing PHC [45]. The review identified 6 types of digital technologies implemented in this context: client-to-provider telemedicine, provider-to-provider telemedicine, targeted client communication, health worker decision support, tracking of patients’ health status, and client participation and self-care. Most studies included were from the United States and Europe, except for one from Argentina.

In terms of implementation outcomes, this review showed consistent findings for cost savings, decreased health care expenses, and improved perceptions among HCPs regarding the quality of the workplace and services offered. However, the results were mixed for other outcomes, such as hospital admissions, readmissions, emergency department visits, the number and cost of drug prescriptions, and patients’ perception (increased satisfaction with timely appointments and self-management support but not with PHC performance). Regarding clinical conditions or behavior change outcomes, there were consistent improvements in blood pressure and glycated hemoglobin control and reduction in amputation, smoking, and the number of patients with end-stage renal disease, but this was not the case for outcomes related to cardiovascular diseases [45].

#### Care Coordination

A systematic review analyzed how digital interventions have supported the laboratory testing process in PHC considering all its phases, from the decision process on the need to carry out a diagnostic test to the interpretation and communication of the test result, contributing to care coordination [46]. The review identified 4 types of digital interventions: health worker decision support, tracking of patients’ health status, client participation and self-care, and laboratory and diagnostics imaging management. Most studies included in this review were from the United States, with some conducted in Europe and Peru, and most of them focused on health service outcomes, but some implementation outcomes were related to health professionals’ perceptions.

The studies included in this review indicated that digital interventions related to tracking patients’ health status and health worker decision support resulted in reducing the amount of effort spent by the staff on collecting information and promoting best care practices but not necessarily reducing the number of diagnostic tests prescribed in the preanalytical phases. The digital laboratory and diagnostics imaging management interventions seemed to impact health care positively in all phases of the testing process, reducing professional workload and the number of errors when the technology was integrated into ambulatory systems and saving time to order prescriptions, reorganize tasks in the intra-analytical phase, and return the test results. Despite this positive impact on health services, in some studies, health professionals indicated that these systems need to be improved in terms of design and reliability, and technical problems and lack of integration between systems were associated with negative effects on the workflow and increased workload. Finally, client participation and self-care interventions to directly communicate the test results to the patient contributed to better collaboration between patients and HCPs and promoted the self-management of chronic diseases [46].

## Discussion

### Principal Findings

This overview of systematic and scoping reviews on digital health technologies applied to PHC revealed the types of technology and the health issues addressed by these technologies that are currently most in evidence in PHC settings. The included reviews focused on technologies related to interventions on client-to-provider telemedicine [30-32], provider-to-provider telemedicine [33], health worker decision support [34-36], tracking patients’ health status [18], client participation and self-care [37,38], and provision of education and training for health workers [39]. There was an expectation that digital health contributes to the improvement of preventive care [40], chronic disease management [41], behavioral health disorder care [42], and delivery of PHC services in pandemic contexts [43,44]. Furthermore, the included reviews indicated the contribution of digital technologies to processes that require coordinating the activities of health workers [45] or different facilities for the provision of health care [46].

Client-to-provider and provider-to-provider telemedicine interventions, previously designed to meet demands from remote or underserved areas, began to be widely used during the COVID-19 pandemic. Social distancing policies spread the implementation of this type of digital intervention as the most viable form of PHC for patients who required continuous monitoring of their clinical conditions. Associated with these technologies, interventions monitoring patients’ health status contributed to the management of patients with chronic diseases in the pandemic context [2].

The emphasis on client participation and self-management interventions is a trend that can be observed in several sectors of the economy. This approach allows companies to lower costs while requiring a more active role from consumers. Through the provision of a patient portal, patients can update personal data, consult and schedule appointments and examinations, check examination results, and obtain clinical history information. By doing so, patients take responsibility for their health care and self-manage their clinical conditions. This type of technology is currently used by companies in other sectors, such as airlines that make travelers responsible for purchasing tickets on a web platform [47] and supermarkets that make customers responsible for scanning their merchandise at self-service checkouts [48]. Similarly, the provision of education and training to HCPs through digital technology follows the trend of distance education in general. Learning software has been widespread since the 2000s, when the internet and PCs became more accessible, and is currently an efficient tool for mass education [49,50].

An important topic that emerged from the reviews included in this overview is the need for providing competency training in computer skills and software use for health workers [34,36,41,43-45], in addition to offering guidance to patients [41]. Nonetheless, these reviews did not mention which skills HCPs and patients need to be trained in to use digital technologies. Neve et al [51] argue that HCPs need to learn to work with and interpret the app-derived data and discuss this information with patients, in addition to being trained in communication skills in digital interfaces and workflow management when there are multiple methods of communication between clients and HCPs. Patients also require assistance and guidance in using digital health technologies. Understanding the PHC worker and patient attitudes and needs for ensuring the adequate use of technologies is critical for their effectiveness [2,51,52].

Training capabilities are even more important when discussing the possibility of delegating duties to other professionals, such as community health workers or nurses. Agarwal et al [36] showed positive results in medication adherence in patients with chronic illnesses when assisted by community health agents supported by computerized CDSS. This discussion is critical, especially in countries with a lack of physicians or underserved areas, and this can be an alternative to improving access to health services in PHC [53].

Some reviews included in this overview (7/18, 39%) also pointed out that adequate use and acceptability across health care workers and patients are influenced by the software or app design, which must be user-friendly and intuitive [18,30,34,38,43,44,46]. Furthermore, technologies must be integrated with systems already used to avoid an increase in the volume of work and rework. Digital health information systems should be designed in line with the functions of PHC. They must also integrate effectively with digital health functions, which include aggregation (patient, diagnostic, and clinician reporting systems and automated collection systems), analysis, and use of information for action [54].

A relevant barrier to the implementation of digital health technologies, mainly those related to client-to-HCP and HCP-to-HCP communication, is the lack of reimbursement and billing strategies for remote consultations [30,31,33]. These new methods of communication between patients and health professionals create new tasks for the latter, which need to be taken into account for payment purposes. These new tasks should also be included in the PHC workflow to avoid excessively increasing the workload. Public policies need to guarantee reimbursement for health care teams for remote care. Research shows that, although this type of policy has been implemented in some countries, results are still mixed in changing clinical practice [2]. Therefore, more research is needed to design reimbursement models that are effective in encouraging the use of this type of digital technology.

The main limitation of this overview of reviews is that most of the included reviews (16/18, 89%) analyzed studies conducted in high-income countries, mainly in North America and Europe. Most of the studies from Europe were conducted in England. The concentration of digital health in high-income countries is a current research concern that was addressed by Xiong et al [41]. They identified 4 main factors that influence digital health service uptake in low- and middle-income countries: governmental regulation and political commitment, the spread of information and communications technology use with interoperability across platforms, user-centered design, and training and incentives for sustainable use of digital tools by health workers and patients. In addition, more research has been conducted to explore this topic, providing insights for developing, implementing, and scaling up digital health initiatives [55,56]; analyzing technologies for improving disease diagnosis and management at the point of care [57]; and mapping digital health interventions for high-risk pregnancies in limited-resource settings [58].

Furthermore, other limitations inherent to the type of methodology used can be highlighted. Overviews of reviews are dependent on the topics covered in published systematic or scoping reviews. Therefore, although this study potentially captured the most relevant types of digital technologies and health issues addressed by these tools in PHC settings, it does not reveal all the possible applications of digital health in PHC. Similarly, overviews of reviews are dependent on the reporting of the included research syntheses. In addition, although the included reviews presented a good methodological quality, this study summarized the results as they were presented by the authors of these reviews. Moreover, relevant studies may have been omitted due to errors in the selection, appraisal, or data extraction processes.

### Conclusions

Finally, despite most studies in the included reviews finding consistent results regarding the implementation of digital interventions, they showed several mixed results related to health service quality and patients’ clinical conditions or behavior changes [36,41,42,45]. Deficiencies in the implementation of the analyzed technologies may have caused those mixed results. Low adherence to digital intervention protocols by health professionals, lack of acceptability by patients, and difficulties in using the software or app can harm the achievement of results at the level of health services and subsequently in patient-level outcomes. Analyzing the results of a digital health intervention requires examining the entire impact pathway, including the linkages among implementation, health service quality, business models, and clinical condition outcomes, which helps identify bottlenecks throughout the process. The theory of change offers a model to describe the causal assumptions behind the links in the pathway, which explains how change is expected to happen [59]. Therefore, those explanations may contribute to digital health implementation in other settings, which can effectively improve health care quality and patient clinical conditions.

## Appendix

Multimedia Appendix 1 PRISMA (Preferred Reporting Items for Systematic Reviews and Meta-Analyses) checklist.

Multimedia Appendix 2 Search strategy.

## Abbreviations

CDSS: clinical decision support system
EHR: electronic health record
HCP: health care provider
PHC: primary health care
PHR: personal health record
PICOS: population, intervention, comparator, outcome, and study design
PRISMA: Preferred Reporting Items for Systematic Reviews and Meta-Analyses
WHO: World Health Organization

## Data Availability

The datasets generated during and analyzed during this study are available from the corresponding author on reasonable request.
