# Supplementary material for: Provision of Digital Primary Health Care Services: Overview of Reviews
Source: J Med Internet Res. 2024 Oct 29;26:e53594. doi: 10.2196/53594 (PMC11558215; doi:10.2196/53594)
Supplement: Multimedia Appendix 2 [file jmir_v26i1e53594_app2.docx]

**Provision of Digital Primary Health Care Services: Overview of Reviews**

**Search strategy**

| **Concept** | **Keywords** |
| --- | --- |
| Digital technologies applied to health | “digital health” OR “e-health” OR “medical informatics” OR “health informatics” OR “health IT” |
| Primary Care | “primary care” OR “primary healthcare” OR “primary health care” |

Search strategy on Web of Science Core Collection (WoSCC)

TS=(“digital health” OR “e-health” OR “medical informatics” OR “health informatics” OR “health IT”) AND TS=(“primary care” OR “primary healthcare” OR “primary health care”)

Search strategy on PubMed/Medline databases from the National Library of Medicine (NLM)

("digital health"[Title/Abstract] OR "e-health"[Title/Abstract] OR "medical informatics"[Title/Abstract] OR "health informatics"[Title/Abstract] OR "health IT"[Title/Abstract]) AND ("primary care"[Title/Abstract] OR "primary healthcare"[Title/Abstract] OR "primary health care"[Title/Abstract])
